# Repressor Element-1 Binding Transcription Factor (REST) as a Possible Epigenetic Regulator of Neurodegeneration and MicroRNA-Based Therapeutic Strategies

**DOI:** 10.1007/s12035-023-03437-1

**Published:** 2023-06-16

**Authors:** Ajmal Nassar, Sairaj Satarker, Prasada Chowdari Gurram, Dinesh Upadhya, SM Fayaz, Madhavan Nampoothiri

**Affiliations:** 1 Department of Pharmacology, Manipal College of Pharmaceutical Sciences, Manipal Academy of Higher Education, Manipal, Karnataka 576104 India; 2 Centre for Molecular Neurosciences, Kasturba Medical College Manipal Academy of Higher Education, Manipal, Karnataka 576104 India; 3 Department of Biotechnology, Manipal Institute of Technology, Manipal Academy of Higher Education, Manipal, Karnataka 576104 India

**Keywords:** Repressor element-1 binding transcription factor, MicroRNAs, Extracellular vesicles, Parkinson’s disease, Alzheimer’s disease, Huntington’s disease

## Abstract

Neurodegenerative disorders (NDD) have grabbed significant scientific consideration due to their fast increase in prevalence worldwide. The specific pathophysiology of the disease and the amazing changes in the brain that take place as it advances are still the top issues of contemporary research. Transcription factors play a decisive role in integrating various signal transduction pathways to ensure homeostasis. Disruptions in the regulation of transcription can result in various pathologies, including NDD. Numerous microRNAs and epigenetic transcription factors have emerged as candidates for determining the precise etiology of NDD. Consequently, understanding by what means transcription factors are regulated and how the deregulation of transcription factors contributes to neurological dysfunction is important to the therapeutic targeting of pathways that they modulate. RE1-silencing transcription factor (REST) also named neuron-restrictive silencer factor (NRSF) has been studied in the pathophysiology of NDD. REST was realized to be a part of a neuroprotective element with the ability to be tuned and influenced by numerous microRNAs, such as microRNAs 124, 132, and 9 implicated in NDD. This article looks at the role of REST and the influence of various microRNAs in controlling REST function in the progression of Alzheimer’s disease (AD), Parkinson’s disease (PD), and Huntington’s disease (HD) disease. Furthermore, to therapeutically exploit the possibility of targeting various microRNAs, we bring forth an overview of drug-delivery systems to modulate the microRNAs regulating REST in NDD.

**Graphical abstract:**

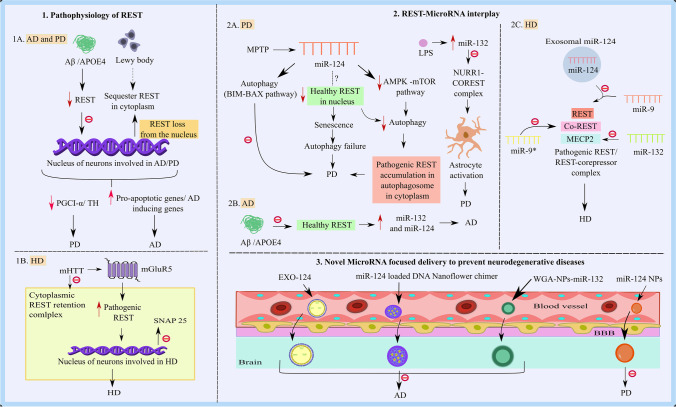

## Introduction

Repressor element-1 binding transcription factor (REST), generally referred to as neuron-restrictive silencer factor (NRSF), is a transcriptional repressor that is typically expressed throughout embryogenesis and is essential for regulating the genes that are unique to neurons. The genes modulating neurotransmitter synthetases, synaptic vesicle proteins, transporters, receptors, and various ion channels in the central nervous system (CNS) are regulated by REST [1–5]. Increasingly evident data support the involvement of REST in epigenetic processes such as the methylation of DNA and the alteration of histones that modulate gene expression dynamics throughout a neuron’s life [5]. One DNA-binding domain and two N and C repressor domains make up the REST protein. The N-terminus of REST recruits its main corepressor, Sin3a, which in turn interacts with histone deacetylases (HDAC-1/2) [5]. However, the C-terminus is accountable for the recruitment of vital corepressors like REST corepressor 1 (CoREST), which successively recruits chromatin remodeling proteins like HDAC-1/2. Numerous investigations have established CoREST and HDAC as essential corepressors of REST. Furthermore, several corepressors are invited by the CoREST/HDAC complex to contribute to the repressive environment around REST targets. Methyl-CpG-binding protein (MECP2), carboxy-terminal binding protein 1 (CTBP1), lysine-specific demethylase 1 (LSD1), and euchromatic histone-lysine N-methyltransferase 2 (EHMT2) or G9a are among them [6].

In the brain, REST is detected more in the hippocampus and cortex but is also seen in the cerebellum, substantia nigra (SNpc), and caudate nucleus [7, 8]. REST levels have also been detected in astrocytes and microglia [9]. Aging is an important factor for REST induction in the brain [10] [11]. REST is an indispensable transcription factor (TF) for mitigating the senescence phenotype in mouse neurons [12]. In the normal aging process, REST is expressed in the nucleus of differentiated neurons where it helps genes be fine-tuned to modulate synaptic plasticity. The deprivation of REST in neurons during aging can contribute to neurodegenerative diseases [13, 14]. In neurons, increased REST expression protects against various cellular damages such as neuronal apoptosis, amyloid beta (Aβ) toxicity, and oxidative stress in healthy individuals [10]. Additionally, it helps to maintain the homeostatic balance of several defender genes (BCL2, FOXO1, SOD1) and degenerative ones (MAPK11, FAS, FADD, TRADD, BAX, BID, DAXX, PUMA, and cytochrome-c) [10]. Despite the neuro-beneficial role of REST in aging, a recent study showed elevated REST in diabetes-induced mouse neurons, and REST knockdown averted the senescence that was brought on by diabetes [15]. Moreover, upon activation of autophagy by oxidative stress in Alzheimer’s disease (AD), this transcription factor translocated from the nucleus to the cytoplasm and is subsequently engulfed by an autophagosome [10]. In the brains of individuals who have Parkinson’s disease (PD) and dementia with Lewy bodies (DLB), REST is generally absent from the nucleus of neurons and is primarily sequestered in Lewy bodies [13]. The development of PD may be linked to the decrease of nuclear REST in senile dopaminergic (DA) neurons [13]. Huntington’s disease (HD) first manifests as an abnormal build-up of REST in the striatal neurons’ nuclei [16, 17].

MicroRNAs (miRs) are a family of gene regulatory elements demonstrated to be crucial in the molecular regulation of brain aging and neurodegeneration [18, 19, 20]. MiRs are non-coding RNA molecules that bind to the 3′-untranslated regions (UTRs) of target mRNAs to stop translation. Despite the lack of definitive evidence that REST affects miRs and their potential function in the CNS, three miRs (miR-124, miR-132, and miR-9) provide a significant clue for the effects of REST interaction with miRs on the CNS [21]. REST regulation by modulation of miRs is a novel research paradigm; the extent to which it contributes to the underlying factors of NDD like AD, PD, and HD is comparably less understood. There remains an unmet demand for clinically beneficial therapies for patients suffering from NDD. Therefore, in our review, we evaluate the interconnection between REST and miRs, especially miR-124 and miR-132, and their roles in PD, HD, and AD. Also, we describe promising strategies for the effective delivery of miRs to the brain, which may broaden the current range of treatments for NDD.

## Huntington’s Disease Pathogenesis and REST

Analysing thirty three studies released between 2010 and 2022, incidence of HD as a group was 0.48 instances per 100,000 person-year [22]. HD is a neurodegenerative condition marked by massive polyglutamine (poly Q) amplification in the huntingtin (HTT) protein’s N-terminus [23]. The HTT protein’s structural characteristics and functional activities are affected by the enlarged CAG repeat, which is translated into longer glutamine at the amino terminus [24]. Chromatin reorganization and transcriptional control are tightly regulated under physiological circumstances. Variations in chromatin remodeling and transcription inefficiency are anticipated to predominate in the causation of HD [25]. Histone acetylation, which is critical for maintaining the correct repressive environment around REST target genes, has a massive effect on the progression of HD [26].

Mutant HTT (mHTT) has a significant impact on epigenetic dysregulation, which leads to several pathogenic outcomes that promote HD [27]. mHTT, a critical pathogenic weapon in HD, was discovered to have a detrimental impact on REST by impairing REST cytoplastic retention, resulting in REST nuclear entry and subsequent REST target suppression. REST nuclear translocation in HD neurons causes suppression of essential genes regulating HD [28, 29]. Paradoxically, nuclear translocation of REST is necessary for healthy aging and reducing AD [10]. As a result, the beneficial and detrimental consequences of REST nuclear translocation in HD and AD neurons, respectively, indicate a fundamental scientific concern about how REST nuclear translocation can have distinct effects on neurons in various circumstances. Furthermore, poly Q expansion disrupted REST’s binding to HTT, which was assisting in the retention of REST in the cytoplasm [16]. HTT gene mutations cause the HTT protein’s N-terminal poly Q repeat expansion [30]. REST appears to be kept in the cytosol via the interaction between HTT and REST, preventing REST target gene suppression. Mutant poly Q-HTT binding to the REST, REST-interacting LIM domain protein (RILP), dynactin, and HTT-associated protein 1 (HAP1) complex changes the complex’s structure, allowing REST to be transported to the cell’s nucleus and contributing to the shutdown of various neuronal genes [31]. Additionally, it has been discovered that poly Q expansion elevated REST gene expression while concurrently preventing embryonic stem cell-derived neurons from developing into neurons [32]. This REST-HTT framework impairment is large enough to cause REST entry into the nucleus, affecting neuronal gene promoters that include critical RE-1 repressor sequences, ultimately leading to their repression. REST also encourages its primary corepressors, including mSin3, CoREST, MECP2, and HDAC-1/2 recruitment to create a robust epigenetic environment around the REST targeting neuronal genes [33]. However, how these corepressors participate in REST’s transcriptional repression remains unknown.

In HD, through the examination of numerous proteins, a distinct and well-defined mechanism for both REST nuclear entrance, as well as REST retention in the cytoplasm, has been established. HTT, RILP, and dynactin p150Glued* interaction modulates REST nuclear transport. HAP1, in addition to this, joins this complex via binding to HTT. Overall complex cooperates to keep REST within the cytoplasm. The capacity of mHTT to dismantle this REST neuroprotection complex causes a nuclear elevation of REST, which offers hints about the crucial mediators regulating REST mobility in HD [31].

HIPP1, also known as huntingtin interacting protein 1 (HIP1) protein interactor, and HIP1, HIPP1’s molecular homolog, tightly control REST and its targets. In addition to acting as the nuclear transporter for HIPPI, HIP1 also infrequently interacts with mHTT. On the other hand, the weaker connections between mHTT and HIP1 considerably increase the massive accumulation of HIPPI and HIP1 inside the nucleus. But HIPP1 enormously binds to the REST promoter and activates it, which in turn represses REST targets [34].

**Fig. 1:**
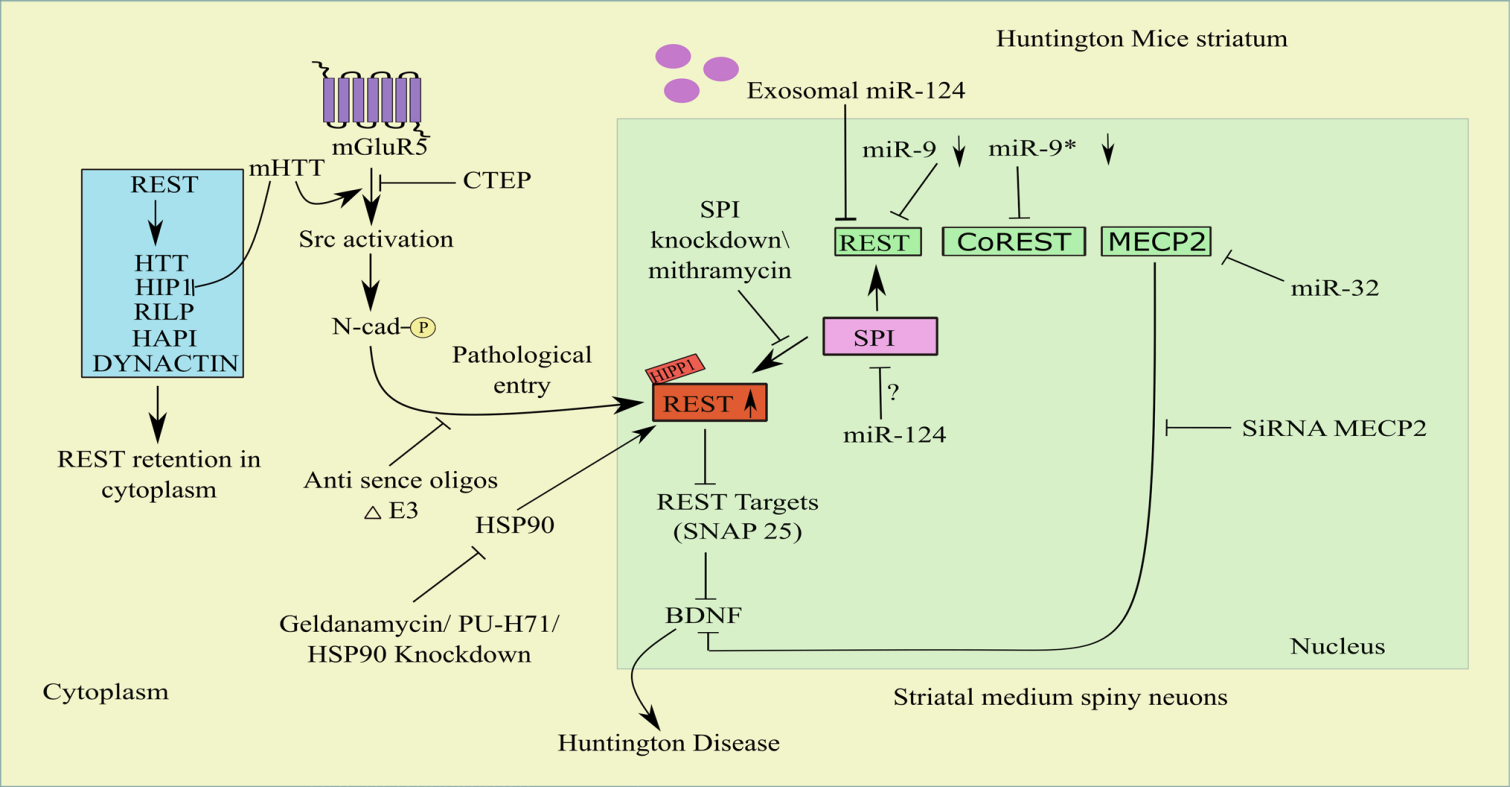
Mechanism of REST nuclear translocation and inhibition of REST nuclear entry/levels by genetic/drug/miRs-based approaches in HD. Collaboration between HTT, RILP, and dynactin p150Glued* modulates REST nuclear transport. HAP1, in addition to this, joins this complex via binding to HTT. The overall complex cooperates to keep REST within the cytoplasm. REST is kept within the cytoplasm by this overall complex. mHTT dismantles this complex, causing a nuclear elevation of REST. The REST promoter is activated, and the REST target genes are repressed by the very weak interactions between mHTT and HIP1, which boost the nuclear accumulation of HIPPI and HIP1. REST’s binding to HTT was disrupted, which was assisting in the retention of REST in the cytoplasm. Elevated Src and N-cadherin phosphorylation via mGluR5 elevation is the root cause of the REST entry and suppression of REST targets such as SNAP-25 and BDNF. Induction of exon skipping (∆E3) in HD cells by producing antisense oligos (ASOS) targeting the splicing locations of REST e3 causes hindering of the REST nuclear entry. REST and mHTT levels are lowered by direct reduction of endogenous Hsp90. REST and CoREST are both highly repressed by miR-9 and miR-9*, respectively. Moreover, exosomal miR-124 targets REST and miR-132 targets MECP2, which in turn inhibits BDNF. Abbreviations: HD, Huntington’s disease; mHTT, mutant muntingtin; poly Q, polyglutamine; HTT, huntingtin protein; RILP, REST/NRSF-interacting LIM domain protein; Dynactin, P150(Glued) subunit of dynactin; HAP1, HTT-associated protein 1; HIPPI, huntingtin interacting protein 1 (HIP1) protein interactor; SNAP 25, synaptosomal-associated protein 25; SP1, specificity protein; ZF, zinc fingers; ∆E3, exon-3 skipping; ASOS, antisense oligos; STMN2, Stathmin-2; SYN1, Synapsin I; miR-9, microRNA-9-5p; miR-9*, microRNA-9-3p

Conversely, REST nuclear entry via metabotropic glutamate receptor 5 (mGluR5) has been established. mGluR5 can efficiently control mHTT-mediated REST dysregulation. HD is induced by pathological elevation of mGluR5 and concurrent aberration of REST [28]. Intriguingly, mGluR5 silencing in HD by genetic and pharmacologic paradigms can reduce these disrupted signaling events and their accumulation. Both synaptosomal-associated protein 25 (SNAP25) and BDNF are REST targets in HD. Its decrement, which occurs because of abnormal REST entry into the nucleus via mHTT-induced pathological mGluR5, has a major impact on the survival of medium-sized striatal spiny neurons. Elevated Src and N-cadherin phosphorylation via mGluR5 elevation is the root cause of the REST entry and subsequent pathological defects of HD [28]. Furthermore, SP1, or specificity protein 1, a REST activator found to be elevated by mHTT, plays an integral role in the pathogenesis of HD. The pathogenic causal events that evolved in HD because of SP1-mediated REST upregulation could be reversed by SP1 knockdown or using an SP1 inhibitor [35, 36] (indicated in Fig. 1).

Since REST tends to undergo alternative splicing events, it can produce a wide variety of mRNAs and proteins. Nuclear REST over accumulation and consequent catastrophic repression of vital neuronal genes helps in maintaining neuronal homeostasis and appears to be highly tied to alternative splicing events. Despite the discovery of 45 new REST-spliced isoforms [37], little is known about their significance. Numerous investigations concentrating on REST4—a REST isoform—showed that REST isoforms have a significant impact on several neurological illnesses. Even though REST4 is devoid of zinc fingers (ZF5) and nuclear localization signals (NLS), its ability to translocate into the nucleus emphasizes the importance of REST-spliced isoforms and their contribution to REST nuclear availability [38]. However, whether REST4 has a pathogenic effect on HD is still a point of contention. REST nuclear targeting is based on exon-3 skipping (E3), which was common in both human and nonhuman primates and is important in the elimination of ZF5, which is the basis for REST nuclear targeting. Surprisingly, induction of ∆E_3_ in STHdhQ111/Q111 cells by producing antisense oligos (ASOS) targeting the splicing locations of REST e3 restores REST targets such as BDNF, Stathmin-2 (Stmn2), and Synapsin I (Syn1) through hindering the REST nuclear entry [29]. REST has recently been introduced to be a target of heat shock protein 90 (Hsp90). Since Hsp90 is necessary for the upkeep of REST and mHTT levels, Hsp90-REST and Hsp90-mHTT interactions have a devastating effect on HD [39] (indicated in Fig. 1).

## The Interplay Between MicroRNAs and REST in HD

The mechanisms that exist between miRs and REST in HD are not clear. The heterogeneity observed in the interaction between REST and miRs could suggest the involvement of different signaling pathways. Some important miRs, such as miR-29a, -29b, -124a, -135b, -139, -203, -204, -212, -330, and -346, have notably shown their involvement in REST expression regulation in addition to miR-9, -132, and -124 [40, 41]. As has already been demonstrated in HD models, miR-9, miR-9*, miR-124, and miR-132 are predicted to upregulate following REST knockdown [21]. Apart from these four miRs, several miRs, however, show a considerable rise. REST is crucial for sustaining the miRs in an in vitro HD model, as demonstrated by the elevated miR-23b, miR-135b, miR-135b*, miR-212, miR-222, miR-153, miR-455, and miR-137 levels. But for three of these enhanced miRs, miR-137, miR-153, and miR-455, the predicted REST attachment sites are 859 bp, 7735 bp, and 859 bp from their corresponding transcriptional start sites, respectively [21]. However, REST knockdown ultimately results in substantially diminished REST availability at miR-137 and miR-153, indicating that these miRs in HD are primarily regulated by REST. The findings of this study showed that a handful of the dysregulated miRs in HD are severely suppressed by high REST levels [21]. Additionally, MiR-22’s pivotal role in the neuroprotection approach by modulating CoREST and histone deacetylase 4 (HDAC-4) was also demonstrated [42]. HDAC-4 which was shown to interact with REST during ischemia [43]. All this vital evidence proves that REST-focused epigenetic repressors participate in HD.

### MIR-124

miR-124 is one of the myriad small non-coding RNAs that is widely investigated for its role in neuronal development and neurodegeneration [19, 20]. miR-124 is neuron-specific and is also expressed in microglia [44]. Furthermore, the most prevalent brain-specific miR, miR-124, is reported to be crucial in controlling adult neurogenesis, synaptic plasticity, neuronal differentiation, neuronal fate, and microglia quiescence [44–48]. miR-124 shows significant alteration in HD and is repressed in HD [49, 50, 51]. Over the past few years, exosomal delivery techniques have greatly improved miR-124 transfer to the brain [52]. Earlier research in the HD model showed that miR-124 downregulation promoted REST upregulation [53]. Recently, exosomes that have been enriched with miR-124 (Exo-124) showed potential to suppress REST in the striatum of r6/2 transgenic HD mice [49]. It was intriguing to see that Exo-124 therapy had no impact on the behavioral improvement of HD animals [49]. Furthermore, miR-124 was downregulated and REST target BDNF was overexpressed in an animal model of HD [54]. Even though miR-124 downregulation and REST overexpression were visible in HD, the mechanism of REST target-BDNF upregulation remains a mystery. This gives information on REST and miR-124’s independent interplay in modulating BDNF and slowing the course of HD.

### MIR-132

MiR-132 downregulation and REST upregulation were demonstrated in HD [53]. A significant reduction in the miR-132 level was observed in HD mice [55]. miR-132 supplementation improves motor performance and delays senescence in HD mice via lowering the brain miR-132 deficit. It is worth noting that miR-132 supplements have no effect on disease-causing mHTT and their targets [55]. The therapeutic potential of miRs has been well-established in HD [56]. Furthermore, miR-132’s target gene, MECP2, a REST corepressor, was discovered to have a high level of expression in addition to miR-132 downregulation in HD mice. Furthermore, mHTT and its considerable aberrant interaction with MECP2 display transcriptional impairment in HD [55]. Addressing the downregulation of REST-influenced miRs and the augmentation of REST reveals a complex interplay between TFs and miRs in HD. However, the involvement of REST cofactors and REST isoforms in the epigenetic framework, as well as their interactions with numerous miRs, should be investigated further in the future.

### MIR-9

REST and its corepressor CoREST is potentially regulated by miR-9 [57]. In HD patients and mouse models, miRs for REST modulation such as miR-9 were downregulated with concomitant upregulation of REST [53]. Additionally, reduced expression of miR-9* has been observed in HD patients’ peripheral leucocytes as a neurodegeneration marker [58]. It is interesting to note that miR-9 or miR-9-5p diminishes the level of REST in HD patients, but miR-9* or miR-9-3p directly contributes to the demise of CoREST [53, 57] (indicated in Fig. 1). CoREST upregulation mediated by miR-9* appears to be involved in HD progression as well [57]. This explicitly states that REST corepressors also have a unique function in the suppression of transcription of their target genes. However, like REST, its corepressors may be affected by miRs. However, more research is needed to confirm how miRs impact REST corepressors.

## Role of REST in Amyloid Pathology in Alzheimer’s Disease

**Fig. 2:**
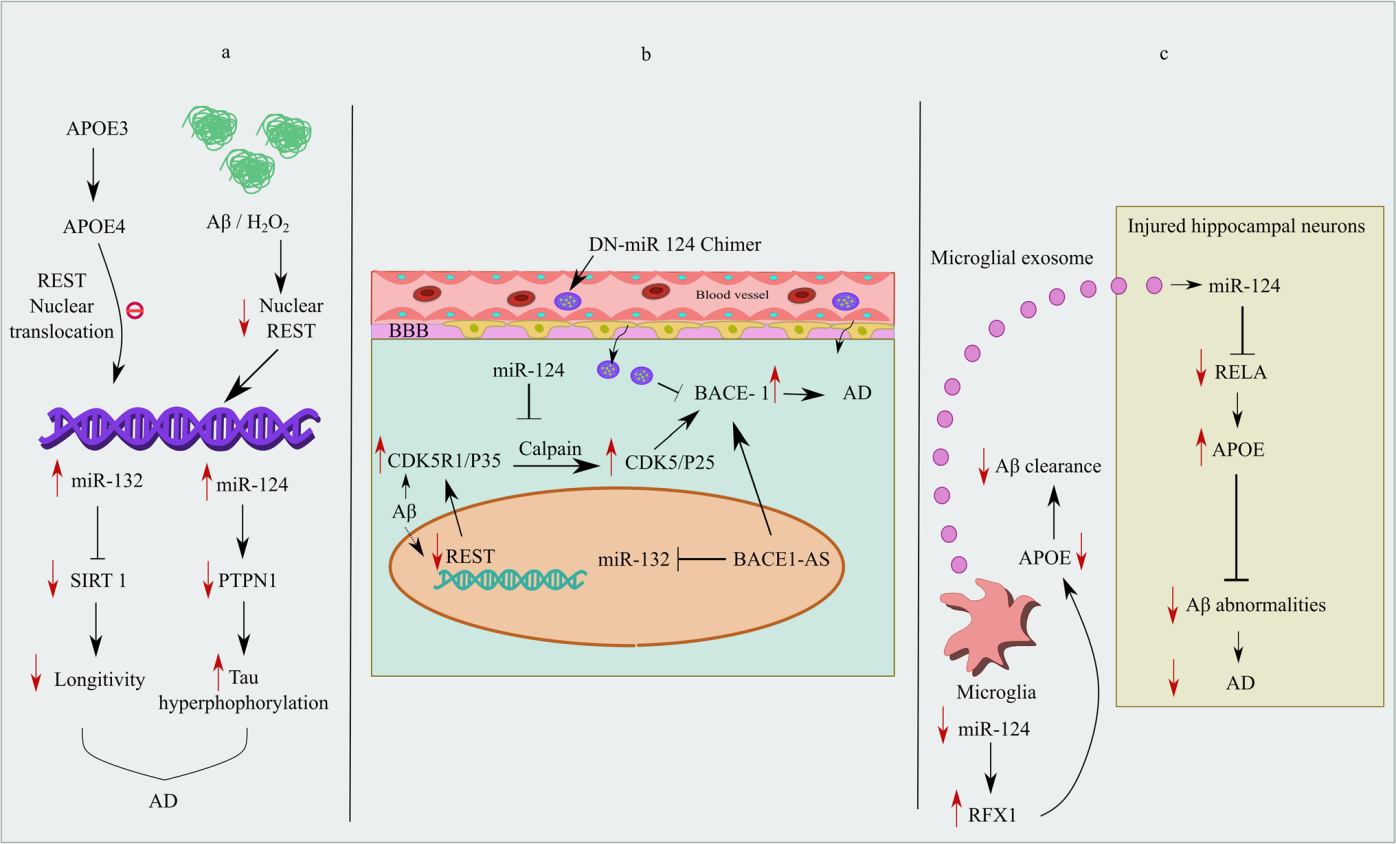
Interplay between REST and miR-132/miR-124 (direct interaction, indirect interaction) and miR-124-APOE interplay in Aβ clearance: plausible involvement of microglia. **a** REST deficiency, direct REST-mediated miR-132, 9 dysregulation, and concurrent miR target deficits in AD (REST loss and concomitant miR-124 upregulation induce AD). In Aβ_1-42_ oligomer (green) treated cells, a decrease in REST occurs and reduced nuclear REST translocation is brought on by the switch of APOE3 to APOE4 expression (blue). In the end, REST failure leads to an increase in miR-124 and a decrease in PTPN1, which in turn encourages tau activation. miR-132 elevation was also shown to be a mediator of Sirtuin 1 (SIRT1) suppression after REST loss, which has an impact on longevity and aging. **b** REST and miR-124/miR-132 both individually influence the CDK5/calpain pathway in AD (REST loss induces AD, miR-124 prevents AD). Aβ_1-42_ induced CDK5R1 overexpression brought on by REST loss was shown. REST loss results in p35/CDK5R1 upregulation, which in turn results in calpain mediated CDK5/p25 activation. Hyperactivated results in BACE-1 phosphorylation and enhanced BACE-1 activity. On the other hand, BACE1-AS stabilizes BACE-1 and initializes BACE-1 genesis. miR-132-3p is inhibited by BACE1 antisense RNA (BACE1-AS). By blocking calpain in a route unrelated to REST action, miR-124 upregulation prevents Aβ_1-42_ driven CDK5R1 overexpression brought on by REST loss and contemporaneous CDK5 activation. miR-124 entry into the brain by DNA nanoflowers mediated miR-124 entry into the brain also suppresses BACE-1 activity and inhibits Aβ genesis. **c** The miR-124-APOE interplay in Aβ clearance: plausible involvement of microglia. Following a mild exposure to hydrogen peroxide, BV2 cells showed a decrease in miR-124 and APOE expression as well as an increase in RFX1 protein level. RFX1 inhibits APOE and impairs Aβ clearance. Microglial exosomes with a prominent elevation of miR-124-3p (Exo-124) result in decreased RELA, which in turn prevents APOE inhibition by RELA and proteolytic breakdown of the amyloid protein in damaged hippocampus neurons. Abbreviations: Aβ, amyloid beta; H_2_O_2_, hydrogen peroxide; APOE, apolipoprotein; CDK5, cyclin-dependent kinase 5; CDK5R1, cyclin-dependent kinase 5 activators 1 (P35); PTPN1, tyrosine-protein phosphatase nonreceptor type 1; Exo-124, microglial exosomes have more miR124-3p; SIRT1, Sirtuin 1; RELA, v-rel avian reticuloendotheliosis; BACE1-AS, BACE1 antisense RNA

There are more than 50 million dementia patients worldwide, according to estimates. Dementia is the fourth biggest cause of disability-adjusted life years (DALYs) lost in those aged 75 and older, and AD is the fifth highest cause of mortality worldwide. Additionally, most patients have one or more family carers, which contributes to psychological morbidity, social isolation, physical illness, and financial difficulty [59]. The most prevalent form of dementia, AD, is brought on by an amalgamation of the pathogenic Aβ creating neuritic plaques and the microtubule-associated protein, tau creating paired helical filament neurofibrillary tangles [60]. Amyloid precursor proteins (APP) are sliced by β-secretase and γ-secretase to form Aβ [61]. The proteolytic cleavage of amyloid precursor proteins (APP) and the production of Aβ could be enhanced by genes such as presenilin 1 (PSEN1) and presenilin 2 (PSEN2), which encipher a major part of γ-secretase [62]. Elevation in the APOE4 allele of apolipoprotein (APOE) enhances the risk and early onset of AD. APOE4 interacts with cytoplasmic tau and leads to hyperphosphorylation [63]. CDK5 is crucial for sustaining synaptic functioning and memory consolidation. The calpain-CDK5 signaling mechanism is triggered by the APOE4 genotype, which results in a boost in site-specific tau phosphorylation [63]. In AD patients, there was a noticeable decrease in the nuclear REST level in neurons, particularly those from the CA1, CA3, and CA4 regions of the hippocampus, as well as prefrontal cortical neurons. However, the neurons of the dentate gyrus and cerebellum did not show a drop in nuclear REST [10]. As compared to cognitively normal control and stable moderate cognitive impairment (MCI) individuals, a significant REST reduction from plasma neuronal-derived exosomes of AD and MCI converting to AD patients was found [64]. Lue and colleagues showed the neuroprotective role of REST in AD by suppressing oxidative stress-mitigating genes and apoptosis-inducing genes that promote amyloid-protein toxicity, while its role in the stimulation of brain oxidative-resistant genes, such as forkhead box protein O1 (FOXO1), indicates that REST could also be a transcriptional activator in the pathogenesis of AD [10]. Furthermore, there is an upregulation of REST repressor function in the brains of individuals with increased longevity, leading to the downregulation of genes that control neuronal excitation and synaptic function. Thus, REST is linked to increased longevity and controls neuronal excitation in the aging brain [65]. Along with suppressing several apoptotic genes such as p38 map kinase MAPK11, FAS, FADD, TRADD, BAX, BID, DAXX, PUMA, and cytochrome-c, REST also targets γ-secretase complex essential components PSEN2 and PEN2, which stimulate Aβ synthesis [10]. Additionally, REST targets include 14-3-3ZETA, a crucial protein for tau hyperphosphorylation, and cyclin-dependent kinase 5 activators 1 (P35 and P39) [10]. REST acts as a protectant factor against toxic insults such as tau phosphorylation and Aβ oligomers in AD through the enhanced expression of the transcription factor FOXO1, which ultimately leads to oxidative stress resistance [10]. The repressive influence of REST on the AD-causing genes speaks of the epigenetic remodeling mechanism crucial to progressive neurodegeneration. But how REST could activate the gene expression of anti-apoptotic factors like BCL2 and antioxidant genes like superoxide dismutase and catalase is still mysterious. The early neuronal changes that cause AD have been studied from a molecular perspective by a specific group, and it was discovered that rather than affecting the entire REST, the translocation defect plays a significant role in regulating sporadic AD (SAD) [66]. The transition of APOE3 to APOE4 results in interruptions in REST nuclear translocation and is the primary source of pathogenic symptoms of AD (indicated in Fig. 2). Surprisingly, after APOE4 induction, REST accumulates in the cytoplasm along with being absent from the nucleus, which facilitates SAD NP cells to differentiate quickly. Reduced nuclear translocation and chromatin binding together with nuclear lamina disruption have been identified as the main consequences of the loss of REST function in neurons caused by SAD [66]. The unanticipated reduction in nuclear translocation of REST following APOE3 to APOE4 conversion further illustrates the drop in the nuclear REST level combined with altered nuclear lamina integrity, which amply highlights the significance of the nuclear REST level [66]. The structural changes in the nuclear lamina are also linked with anomalous tau phosphorylation, CDK5 activation, and altered phospholipid metabolism [67, 68]. How miRs are involved in the regulation of REST in AD, even though REST failure significantly contributes to the dysregulated gene expression in AD neurons, remains to be investigated.

## The Interplay Between MicroRNAs and REST in AD

### MIR-124

One of the key events in the pathophysiology of AD is the downregulation of REST and enhanced expression of miR-124 in the brain and reducing miR-124 expression could be a possible future intervention to be focused on the prevention and management of AD [69]. On the contrary, several investigations also indicate miR-124 downregulation in the pathogenesis of AD [70, 71]. miR-124 regulates APP splicing, thereby causing alterations in the APP gene expression and leading to the production and deposition of Aβ plaques [72]. The tyrosine-protein phosphatase nonreceptor type 1, often known as PTPN1, is a potential miR-124 direct target. miR-124 binds to the 3′-UTRs of PTPN1 and inhibits its translation [69]. Aberrant miR-124-PTPN1 signaling disrupts the α-amino-3-hydroxy-5-methyl-4-isoxazolepropionic acid (AMPA) receptor trafficking, resulting in dysregulated glutamate neurotransmission, synaptic dysfunction, and memory impairment [73]. Amyloid pathology causes the loss of REST, resulting in the elevation of miR-124 and its interaction with PTPN1, causing hyperphosphorylation of tyrosine sites on glycogen synthase kinase 3 (GSK-3-β) and protein phosphatase 2A (PP2A), resulting in an imbalance between tau kinases and phosphatases, leading to the progression of tau pathology [69]. In Aβ_1-42_ oligomer-treated cells, overexpression of REST suppressed the elevation of miR-124 and caused an increase in PTPN1, indicating that loss of REST may mediate the miR-124-PTPN1 signaling in AD (indicated in Fig. 2).

The protein encoded by gene p35/CDK5R1, a REST target gene [10], activates CDK5. Calpain proteolytically pierces the p35/CDK5R1 protein, resulting in the generation of hyperactive CDK5/p25 which in turn triggers tau phosphorylation [74]. Rather than acting as the direct modulator of REST, miR-124 also has the potential to behave in a REST-independent manner. Aβ promotes REST loss and induces the elevated p35/CDK5R1 expression. miR-124 targets calpain (CAPN). miR-124 overexpression mediated CAPN blockade reduced p35/CDK5R1 overexpression induced by REST loss and concurrent CDK5 activation. Hyperactivated CDK5 also results in BACE-1 phosphorylation and enhanced BACE-1 activity [75]. Surprisingly, BACE 1 activity was inhibited by miR-124 and inhibited Aβ generation [70, 76] (indicated in Fig. 2).

### MIR-132

One of the most prevalent miRs in the brain, miR-132 has a significant role in synaptic plasticity and neuronal morphogenesis [77]. In the mouse hippocampus, lack of miR-132 boosts the progression of AD via augmenting critical tau hyperphosphorylation and Aβ generation [77]. Several evidences provide the clue that miR-132 is considerably diminished in AD [78, 79]. In contrast, a few studies also demonstrated enhanced activity of miR-132 in AD [80]. A recent study found that miR-132 declines in dying neurons, possibly because of REST overexpression [81]. Furthermore, miR-132 elevated expression in the brains of AD patients impacts the REST targets such as BAX and CDK5 and suppresses BCL2 [80]. These findings shed light on the REST-mediated miR-132 overexpression in the context of AD. Furthermore, miR-132 mediated Sirtuin 1 (SIRT1) inhibition [82] (indicated in Fig. 2) and diminished REST levels in AD patients [10] have also been proven to have a substantial impact on longevity and aging. FOXO1, a well-known longevity gene [83], also appeared to be a potential governing interactor of miR-132 [84]. Interestingly, REST also acts on FOXO1 in the case of AD as an anti-oxidative stress defense mechanism [10]. It is noteworthy to note that a recent study indicated that transgenic tau-expressing AD mice’s hippocampal levels of miR-132 were constant, even though REST levels were drastically reduced [69]. However, the mystery surrounding REST-miR-132-mediated neurodegeneration remains unsolved. Besides, miR-132-3p is one of the REST-impacted miRs, found to be inhibited by BACE1 antisense RNA (BACE1-AS). BACE1-AS mainly regulates BACE1 expression by enhancing BACE1-mRNA stability and generating more BACE1 through various mechanisms [85]. Critical regulation of the BACE1-AS/miR-132-3p axis could be investigated as a possible therapeutic approach for AD.

## The miR-124/132-APOE Interplay in AD: Plausible Involvement of Microglia

Microglial exosomes have a dominant role in the mobility and distribution of Aβ [86]. In addition to these functions, the ability of microglial exosomes to eradicate Aβ makes them a target in terms of AD therapeutic approaches. Microglia activation and neuroinflammation are two mechanisms via which miR-124 contributes to the pathophysiology of AD [86]. Reduced proteolytic clearance of soluble Aβ in microglia may contribute to Aβ accumulation in AD and its progression [87]. miR-124 appears to have various roles in controlling APOE signaling in AD. After modest hydrogen peroxide exposure on BV2 cells showed a substantial drop in miR-124 rather than an increase, a concurrent decline in APOE expression, and an increase in regulatory factor X1 (RFX1) protein level. It was discovered that RFX1 was notably expressed in microglial cells as well as the nuclei of neurons, pointing to a potential role for RFX1 in microglial cells [88]. RFX1 may be one of the future targets for AD because of its capacity to impede APOE and hence cause a reduction in Aβ clearance [89]. There is an RFX1 binding site in the first intron of the APOE gene and a miR-124 binding site in the 3′-UTR of RFX1 mRNA. Additionally, interrupting this signaling pathway by silencing RFX1 significantly elevated the uptake of Aβ in BV2 cells. These results emphasize the mechanism by which diminished miR-124 production under oxidative stress hindered the uptake of Aβ, implying that RFX1 may be a potential target for enhancing Aβ clearance as we age. APOE encourages the proteolytic breakdown of Aβ, thereby prevents amyloid anomalies [90]. Microglial exosomes having augmented miR-124-3p (Exo-124) prevented Aβ irregularities in hippocampal injured neurons by targeting RELA or v-rel avian reticuloendotheliosis, which is a prominent APOE suppressing TF [87] (indicated in Fig. 2).

It is intriguing that only a small number of studies have been done to describe the function of miR-132 in the microglial cell population and that there is currently no evidence linking miR-132 to APOE directly. In fact, APOE was not even predicted to be a direct miR-132 target until recently. However, there is a strong negative correlation between the levels of miR-132 and APOE across the board in the microglial population. When miR-132 is overexpressed, APOE exhibits considerable downregulation and maintains the homeostatic condition of the microglia, while miR-132 knockdown in microglial cells has been demonstrated to have higher levels of APOE and activate the microglia [91].

## Interaction Between Dopaminergic Neurons and REST in Parkinson’s Disease

According to the Global Burden of Disease (GBD) survey, there were 1.02 million new cases of PD in 2017. Globally, 6.1 million cases of PD were reported in 2016, and the age-standardized rate (ASR) of prevalence rose by 21.7% during the same year. Years lived with disability (YLDs) is a measure of both the handicap brought on by that status and the average time it takes for incident instances to recover or pass away. YLDs is a popular metric for measuring the health damage brought on by PD. From 1990 to 2007, the age-standardized rates of YLDs caused by PD sharply climbed to 8.9%, and they continued to rise between 2007 and 2017. According to studies, the burden of PD would significantly increase in the coming decades [92, 93]. PD occurs due to the aggregation of insoluble α-synuclein in intracellular deposits known as Lewy bodies in the midbrain, striatum, and other regions of the brain leading to a neurodegeneration phase manifested as resting tremor, bradykinesia, rigidity, and postural imbalances. PD produces synucleinopathy, which arises via dopaminergic neuron loss in the SNpc [94]. Although α-synuclein’s physiological role has not yet been fully characterized, it has been connected to DA metabolism and synaptic plasticity. When α-synuclein builds up, it negatively affects mitochondrial dynamics and homeostasis, and neuronal death results from malfunctions in cellular clearance systems such as autophagy and the ubiquitin-proteasome system [95, 96]. In middle-aged subjects (up to 61 years old), REST was found in the cytosol but was not detected in the nucleus. However, in elderly cases (72–81 years old), the presence of REST was found in both the cytosol and nucleus [13]. An interesting feature of the REST-DA interaction in favoring neurodegeneration is the disrupted translocation of REST from the cytosol into the nucleus Hence, a big aspect of maintaining a healthy homeostasis is the entry of REST into the nucleus of DA neurons during aging. In aged neurons, neuronal REST accumulation acts as a neuroprotective mechanism against defective cellular homeostasis. REST is moderately sequestered in Lewy bodies in DA neurons in brains with PD and DLB, where it is absent from the nucleus of neurons. Even though deficits of nuclear REST availability, its depletion also contributes to PD pathogenesis [13].

**Fig. 3:**
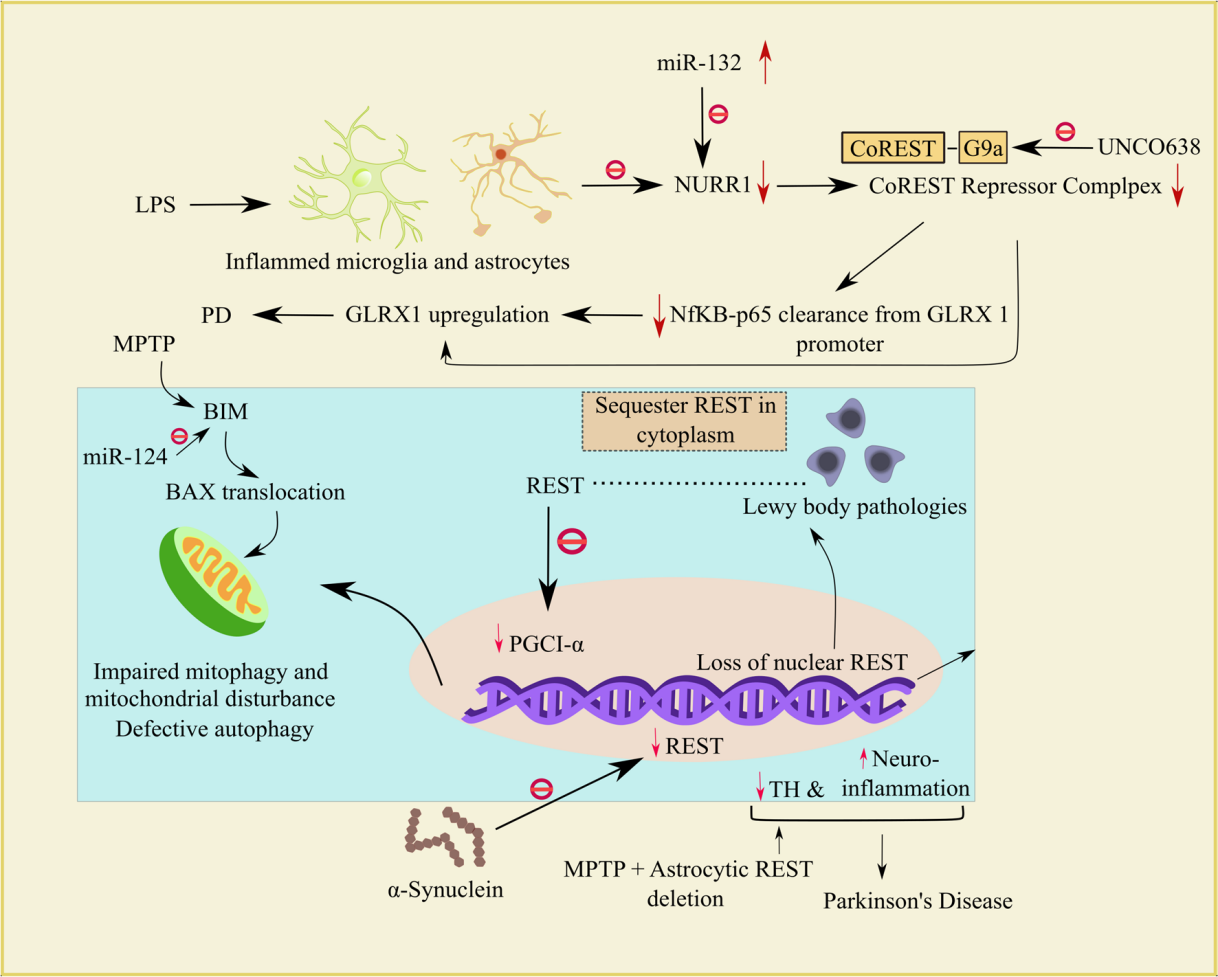
Mechanisms underpinning how REST and miRs interact in PD.REST is typically produced within the nucleus of DA neurons (violet) and inhibited by α-synuclein (brown). Further Lewy bodies (black) disrupt the nuclear accumulation of REST in the dopaminergic neurons and sequester REST in cytoplasm. The impairment of REST ultimately results in the decrement of PGC1-α, which in turn induces faulty mitochondrial function and impairs mitochondrial function and leads to PD (green). miR-124 blocks faulty mitochondrial function by blocking BIM and concomitant BAX translocation to mitochondria. When LPS causes inflammation during PD, the NF-kB-P65 subunit binds to the GLRX1 promoter and suppresses it. However, because miR-132 mediated NURR1 loss during inflammation cannot enlist CoREST and because transcription repression is compromised, NF-kB-p65 on the GLRX1 promoter is not removed as rapidly, resulting in GLRX1 overexpression and PD. REST knockout from astrocytes along with MPTP boost PD associated effects such as decrement in TH level and increase in neuroinflammation. Additionally, UNC0638, an inhibitor of G9a (a REST/CoREST corepressor), promotes GLRX1 overexpression by hindering the NURR1 and CoREST-mediated suppression of GLRX1. Abbreviations: DA, dopamine; PGC1-α, peroxisome proliferator-activated receptor-γ coactivator; miR-124, microRNA-124; BAX, BCL-2-like protein 4; LPS, lipopolysaccharide; GLRX1, glutaredoxin-1; CoREST, REST corepressor 1; BDNF, brain-derived neurotrophic factor; NURR1, nuclear receptor-related 1 protein; NF-kB, nuclear factor kappa-light-chain-enhancer of activated *B* cells; PD, Parkinson’s disease; TSA, Trichostatin-A; TH, tyrosine hydroxylase; miR-132, microRNA-132

Peroxisome proliferator-activated receptor-γ coactivator also known as PGC1-α, a TF that has a distinct responsibility in preserving cellular energy metabolism and they are critically associated with mitochondrial biogenesis. PGC-1α is a potential therapeutic target for PD [97, 98, 99]. PGC1-α has become a promising platform for PD therapy as it acts as a key modulator of mitochondrial biogenesis and cellular antioxidant defense, with a prominent effect on dopaminergic neuron function and survival in the SNpc region [98]. Recent research has demonstrated that REST is an essential element of neuroprotection. In DA neurons lacking nuclear REST, α-synuclein oligomers induce faulty mitochondrial function and impair mitophagy. The overexpression of alpha-synuclein in DA neurons (SNCA-OVX) results in the depletion of both REST levels and PGC1-α, which simultaneously activates mitochondrial dysfunction. Increased expression of REST results in a substantial increase in the PGC1-α level and attenuates mitochondrial damage and mitochondrial morphology alteration [99] (indicated in Fig. 3). The unexpected activation of REST in GABAergic neurons upon SNCA-OVX and prominent ability of GABAergic neurons to prevent α-synuclein oligomers and prevent the reduction of PGC1-α are evidence that the alpha-synuclein-REST-PGC1-α-mediated pathway is different in distinct types of neurons [99].

In DA biosynthesis, tyrosine hydroxylase (TH) acts as a rate-limiting enzyme. REST shields DA neurons against Mn-induced fall in TH level and DA neuron damage. It has been demonstrated that in DA neurons, REST binds to the RE1 consensus sequence in the TH gene promoter. The binding of REST to the TH promoter upregulates TH expression due to the recruitment of cAMP-response element-binding protein. Thus, REST protects the DA neurons from toxic insults that lead to neurodegeneration [100]. On the contrary, REST’s potential to suppress TH transcription has also been reported. A mutation in the RE-1 element region of the TH promoter boosted TH transcription by seven times in human neural stem cells, but no such results were seen in differentiated neural-like cells, implicating the REST function may be different in differentiated cells [101]. In the mature DA neurons, REST’s propensity to trigger TH transcription in the presence of the RE-1 element in the TH promoter, on the other hand, raises the question of whether REST functions in a context-dependent manner rather than always acting as a repressor with the help of miRs.

Activated astrocytes have a crucial role in triggering neuroinflammation [18, 102], a hallmark of PD. Neuroinflammation has long been considered a downstream response to the death of DA neurons. Most studies indicate α-synuclein as an exogenous astrocyte stimulator. α-Synuclein aggregates have been observed in astrocytes alongside neurons in post-mortem PD brains, indicating that astrocytes endocytose α-synuclein secreted from neurons and trigger neurodegeneration along with inflammatory responses [103, 104]. Astrocyte dysfunction has been linked to various pathogenic processes in PD. Hence, restoration of astrocytic function is becoming a target for reversing neurodegeneration and enhancing neuron survival [105]. Moreover, astrocyte-specific conditional knockout of REST in WT mice elicited PD-like behaviors characterized by the loss of DA neurons and a fall in striatal TH levels [106]. The activation of astrocytes and microglia causes the release of pro-inflammatory mediators, triggering neuroinflammation [107, 108] (indicated in Fig. 3). Specific depletion of REST in the brain leads to neuroinflammation along with concomitant impairment of neurogenesis [109]. Inflammatory cytokines such as IL-6, IL-1, and COX-2 translation were increased in REST-deficient astrocytes challenged with lipopolysaccharide (LPS) [109]. Gliosis, which is caused by the inflammasome nucleotide-binding oligomerization domain-like receptor pyrin domain-containing 3 (NLRP3), contributes to PD. The NLRP3 is a crucial part of the defense system that aids in causing caspase-1 activation and the release of pro-inflammatory cytokines like IL-18 and 1L-1β. LPS enables the NLRP3 inflammasome to become active, and LPS can bind to TLR4 and promote the migration of NLRP3 along the NF-kB pathway [110, 111]. The primary pathophysiology of PD is aberrant alpha-synuclein aggregation, which also activates NLRP3 inflammasomes. In animal models, NLRP3 pathway knockdown mitigated DA neuron loss and motor impairment [112]. Through suppressing NF-kB and the NLRP3, DA also promotes neuroprotection via the A2 astrocytic pathways and blocks the A1 astrocytic route in addition to enhancing the production of BDNF and GDNF [113].

HDACs function as an epigenetic corepressor of REST [5]. Trichostatin-A (TSA), a HDAC inhibitor, works in concert with REST to protect neurons in the MPTP model of PD [114]. BDNF was a well-proved REST target [115] [34]. A single dose of TSA increased the levels of REST-influencing genes such as BDNF and TH and protected the nigrostriatal dopaminergic system against MPTP-induced degeneration. Neuroprotection of TSA in PD is stopped by the neuron-specific knockout of REST. Moreover, HDAC inhibition by TSA and concomitant upregulation of key REST targeting genes such as BDNF and TH against MPTP instigated degeneration-triggered mice, implicating the epigenetic involvement of HDAC in the control of REST and REST target genes in PD [114]. In fact, despite the functional distinctiveness, REST-miRs may potentially interact to contribute considerably to the deregulated gene expression in PD neurons.

## The Interplay Between MicroRNAs, REST, and REST Corepressor (CoREST) in PD

REST and miRs play a significant role in HD and AD, and our review has discussed the crucial evidence supporting this. However, there is still no proven data on how directly REST interacts with several miRs in PD. The direct link between the corepressor of REST (CoREST) and miR-132 in PD has been established. It offers vital hints about REST’s involvement in PD and in modifying the REST-associated pathways where CoREST was involved. The role of miR-124 with one of the REST-impacted processes, autophagy, needs to be discussed. The impact of autophagy on PD is substantial. REST, or the miR-124, and autophagy have a well-established link.

### MiR-124 and REST in Autophagy Modulation

Impaired protein homeostasis and the accumulation of misfolded or incorrectly altered proteins are common disease processes in many neurodegenerative diseases, including PD. One of the primary pathways for degradation, autophagy, is essential for maintaining cells’ ability to efficiently turn over proteins and damaged organelles [95]. Dysfunction in autophagy is tied to the occurrence of PD [116]. Any abnormality in intrinsic autophagy activity causes an overabundance of misfolded α-syn, which may then impede their own degradation, starting a cascade of events that ultimately kills neurons [117].

In MPTP-treated neurons, miR-124 overexpression inhibits autophagy, while miR-124 suppression increases and decreases the levels of the two primary autophagy modulators, p-AMPK, and p-mTOR, respectively. So, in DA neurons, miR-124 regulates the AMPK/mTOR pathway, which is involved in cell death and autophagy [118]. The involvement of the BH3-only protein (BIM) and cell death mediator BAX in mitochondrial homeostasis has been observed. BIM is involved in both anti-autophagy as well as proapoptotic processes [119]. Nevertheless, BAX translocation to mitochondria and followed by BIM elevation does have a crucial impact on SNpc DA cell death. It has been demonstrated that miR-124 decreases apoptosis, autophagosome accumulation, and lysosomal depletion by targeting BIM [120]. In MPTP-treated mice, overexpression of miR-124 resulted in decreased BIM activity, BAX translocation inhibition, and hence neuroprotection from MPTP [120] (indicated in Fig. 3). So, this evidence critically demonstrated that miR-124 modulates autophagy in PD.

Nevertheless, autophagy is also a crucial mechanism for PD-related REST regulation. The autophagy-lysosome machinery is used to break down REST. Under typical circumstances, neuronal REST may be constitutively destroyed via the p62-mediated selective autophagy mechanism [13]. REST buildup in clusters in the cytoplasm and removal from the nucleus are both harmful. Mice that are deficient in the autophagy specific to DA neurons have been demonstrated to accumulate REST in TH-positive neurons [13]. Yet, it was shown that REST has an intriguing pathogenic identity that causes it to accumulate in autophagosomes along with pathologically misfolded α-synuclein [10]. Aging is the main risk factor for the beginning and development of PD [121]. Autophagic flux impairments have been observed in PD, which contributes to senescence acquisition. Moreover, an aging brain usually presents with low levels of autophagic activity, with decreased ability to prevent waste accumulation [122, 123]. REST interacts with the autophagic machinery directly, contributing to cellular senescence, and REST suppression inhibits autophagy and mitochondrial function in neurons [12]. Moreover, autophagy failure, loss of control of proteostasis maintenance, augmented oxidative stress, and an accelerated rate of cell death are all consequences of REST deficiency in neurons [12]. The establishment of a reduced REST level by genetic-focused approaches culminates in the emergence of a senescence phenotype by inducing the overexpression of vital inhibitors of cyclin-dependent kinases p21 (CDKN1A) and the lowering of cell viability [12]. Finally, inducing autophagy in REST-deficient neurons improves p21 expression and mitochondrial activity, alleviating the senescence phenotype [12]. Yet, a different group of researchers claimed that a decline in REST had a favorable impact on autophagy. REST was markedly elevated in a diabetic-induced neuronal senescence environment where autophagy was impaired. REST downregulation can impact neuronal aging by inhibiting mTOR and boosting autophagy [15]. In contrast, REST deficiency causes a decrease in autophagy and cellular senescence in neurons. REST deficit boosts and suppresses autophagy. These conflicting results demonstrate that REST controls autophagy in distinct circumstances where neuronal senescence is a key player. In addition, these findings strongly suggest a mutually reinforcing feedback process between REST dysfunction and autophagy dysfunction, where REST suppression causes autophagy modulation and vice versa, while autophagy dysfunction results in REST accumulation with pathogenic misfolded proteins under neurodegenerative conditions.

### MiR-132

The role of miR-132 in neurons and neurodegeneration underlying PD is supported by the evidence that miR-132 is upregulated in PD patients [124]. A rise in miR-132 level was found to be associated with different PD models [125, 126]. Neuroinflammation triggers neurodegeneration, and microglial cells play a key part in this process [127, 128].

Nuclear receptor-related 1 protein (NURR1) is a pivotal TF that plays a key role in strengthening DA neuron functions and ameliorating neuroinflammatory conditions in the CNS. NURR1 acts as an important defender in PD by reducing the production of extremely neurotoxic inciting mediators by astrocytes and microglia [129]. TH-expressing neurons die because of astrocyte-amplified inflammatory reactions that are sparked by the loss of NURR1. The activation of NURR1 limits the inflammatory effects of LPS and the loss of TH+ neurons through the activation of the CoREST complex, followed by the removal of NF-kB-p65 from the GLRX1 promoter and concurrent transcriptional suppression. miR-132 has a strong relationship with DA neuronal death through NURR1 and safeguards against the degeneration of DA neurons in PD [124].

miR-132 controls the expression of NURR1 in the conversion of embryonic stem cells to DA neurons [130] and PD patients [124]. MPTP treatment in astrocyte specific REST cKO mice leads to the activation of astrocytes and microglial cells and affects the nigrostriatal dopaminergic pathway. Lack of REST significantly increases the expression of the key pro-inflammatory markers IL-1, IL-6, COX-2, and IL-17 in microglial cells treated with LPS [109]. Increased microglial and astrocyte activation in PD may be due to loss of REST/CoREST with miR-132 upregulation causing NURR1 suppression. Glutaredoxin (GLRX) is a protein that contributes to the glutathione-dependent disulfide oxidoreduction processes [131]. Miller et al. found that overexpression of LPS-associated GLRX-1 upregulation causes neuroinflammation in mouse and human brain samples to stimulate DA loss in PD [132]. Normally, the microglia’s NURR1-CoREST complex suppresses GLRX1. However, after exposure to LPS, the P65 subunit of the NF-kB attaches to NURR1 and causes it to disengage from the GLRX1 promoter, upregulating GLRX1. Additionally, UNC0638, an inhibitor of G9a (a REST/CoREST corepressor), promotes GLRX1 overexpression by hindering the NURR1 and CoREST-mediated suppression of GLRX1. Upregulation of GLRX1 causes microglia to produce cytokines and a decrease in glutathionylated intracellular proteins. An increase in the release of cytokines is thought to be the catalyst for the death of nearby neurons and results in PD. This strongly shows that CoREST and its related corepressor G9a are necessary to maintain the NURR1-GLRX1 axis during neuroinflammation [132]. Moreover, contradictory results have been reported that GLRX1 deficiency has contributed to neurodegeneration [133] and PD-dependent motor defects [134]. Furthermore, GLRX overexpression in PD patients reverses miR-132-3p-mediated microglial activation and neuroinflammation [135] (indicated in Fig. 3).

## Recent Advances in MicroRNAs-Focused Delivery in Neurodegeneration

### Brain-Drug Delivery Systems to Target miR-124 and miR-132 in AD

Given their high instability and propensity to be broken down by plasma nucleases, miRs are difficult to deliver to the brain and have a low bioavailability. Additionally, because of their negative charge, miRs are unable to pass through the lipid bimolecular layer that forms the cell membrane. Therefore, the effectiveness of miR-mediated therapy depends on choosing a safe and effective delivery method [136]. Vectors with and without viruses have been created to increase the effectiveness of miR delivery. miR-124-3-P delivery through an intracerebral infusion route using an AAV-conventional vector reduced Aβ deposition and facilitated learning and memory in APP/PS1 mice [137]. Additionally, new techniques such as DNA nanoflowers (DFs) have been developed to effectively transfer miRs to the brain. From a designer template, long DNA building blocks are made by rolling circle replication to develop DFs. Due to dense DNA packaging, DFs are resistant to denaturation, dissociation, and nuclease degradation. Recently, miR-124 delivery using DFs reduced BACE1 and APP, thereby preventing Aβ generation in the hippocampus of APP/PS1 mice [76]. This nanosystem-based therapy promotes the long-term availability of miR-124 as well as its blood-brain penetration and subsequent neuron targeting. The DFs are further modified with the rabies virus glycoprotein (RVG29) to achieve BBB penetration and neuron targeting concurrently [76]. Furthermore, microglia-focused miR delivery is also a new strategy to transport miR to the brain [138]. EVs are currently being investigated as a crucial means of transferring messages between cells [139, 140]. Growing data suggest that soluble toxic Aβ peptides in AD are transported, distributed, and cleared by microglial extracellular vehicles (EVs) [141]. Microglial EVs were able to transport the soluble form of the neurotoxic Aβ in AD [142]. The revelation that microglial EVs facilitate the mobility of miRs was an intriguing discovery. Microglia cells have an incredible ability to be transfected by miR-124-3p mimics, which then results in the formation of miR-124-3p enriched microglial exosomes (Exo-124). The potential of Exo-124 to decrease the negative effects of Aβ has been verified and speeds up the reversal of neurodegeneration through a unique, understudied RELA-APOE route [87].

Numerous studies have emphasized the importance of miR-132 as a therapeutic approach to prevent AD. Besides, extremely strong miR-132 expression driven by a lentiviral-mediated transfection tactic drastically reduces Aβ load and overcomes spatial memory deficits in APP/PS1 mice, particularly in the hippocampus [143]. Additionally, miR-132 delivery using plasmids lowered sevoflurane-induced cognitive impairment by suppressing FOXA1, a key TF associated with AD [144]. miR-132 inhibitor-loaded EVs in the brain and the presence of miR-132 inhibitor in the brain draws significant attention to the therapeutic use of EVs in miR-focused perspectives. It has been demonstrated that neuron-derived small EVs (sEVs) maintain neurotransmitter homeostasis and that sEVs may influence Aβ toxicity in neurons. The neuronal cell death that occurs in AD is largely influenced by glutamate excitotoxicity and γ-aminobutyric acid or GABAergic neuron malfunction [145]. The sEVs secreted from neurons treated with GABA reduced the damage induced by Aβ, whereas those released from neurons treated with glutamate increased Aβ toxicity. miR-132 expression was downregulated in glutamate-treated neurons’ sEVs while it was raised in GABA neurons’ sEVs. It is crucial to highlight that GABA-treated neurons exemplify the propensity to increase the release of sEVs enriched with ample miR-132 and show a great capacity for neuroprotection, whereas glutamate-treated neuronal sEVs significantly diminished the level of miR-132, perhaps amplifying the consequences of Aβ toxicity [145]. Furthermore, employing an intravenously administered synthetic miR-132 mimic oligonucleotide in adult mouse AD hippocampi was demonstrated to reverse hippocampal neurogenesis and memory deficits [146].

**Fig. 4:**
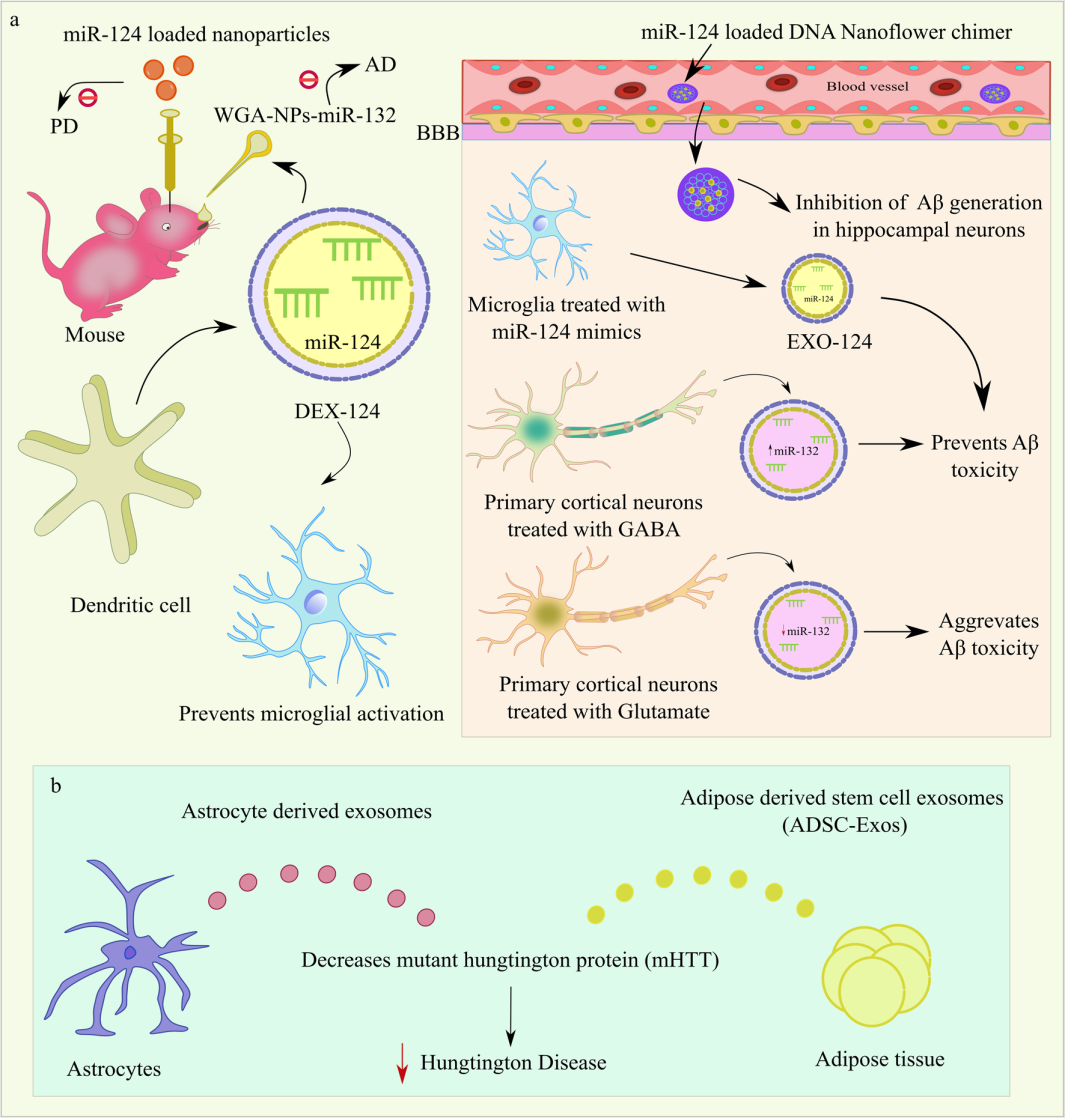
Advanced techniques of miR delivery to prevent AD, PD, and HD. **a** DNA nanoflowers (DFs) promote the long-term availability of miR-124 in circulation as well as its blood-brain penetration and subsequent neuron targeting in AD. Exosomes (Exo-124) from microglial cells exhibit increased miR-124 and reduced Aβ toxicity. Neuron-derived small EVs (sEVs) secreted from neurons treated with GABA raise the miR-132 abundance, whereas lowered miR-132 levels in the glutamate-induced neuronal secretion of sEVs potentially increase Aβ toxicity. Intra-nasal delivery of miRs coated with polymer nanoparticles modified by lectins and wheat germ agglutinin (WGA)-modified PEGPLA nanoparticle with miR-132 (WGA-NPs-miR-132) prevents AD. Through the intranasal route, DEX-124 can be functionally delivered to microglia and prevent their activation. Intracerebroventricular (i.c.v.) administration of biocompatible and trackable polymeric nanoparticles (NPs) loaded with miR-124 en route to the striatum ameliorates PD. **b** Injection of exosomes derived from astrocytic and adipose-derived stem cells into the HD environment prevents mHTT and HD progression. Abbreviations: DFs, DNA nanoflowers; sEVs, neuron-derived small extracellular vesicles; WGA-NPs-miR-132, wheat germ agglutinin (WGA)-modified PEGPLA nanoparticle with miR-132; DEX-124, extracellular vesicles produced from dendritic cells; ADSC-Exos, adipose-derived stem cells exosome enriched with miR-124; BBB, blood-brain barrier

A team of researchers recently investigated the nasal delivery of miRs coated with polymer nanoparticles modified by lectins and wheat germ agglutinin (WGA)-modified PEGPLA nanoparticle with miR-132 (WGA-NPs-miR-132), which demonstrated tremendous potential for neuroprotection in AD [147]. WGA-NP reaches the lamina propria by transcellular pathways following intranasal delivery before being transported to the olfactory nerve bundle or the surrounding connective tissue. Also, a significant number of WGA receptors are present on the surface of neurons in the brain, allowing for efficient WGA absorption by neurons [148] (indicated in Fig. 4).

### Brain-Drug Delivery Systems to Target miR-124 in PD

Recent research has shown that exogenous miR-124 delivered by a viral vector with emphasis on the SNpc of mice treated with MPTP efficiently reduces the activation of microglia by inhibiting the expression of sequestosome 1 (p62) and phospho-p38 mitogen-activated protein kinases (p-p38), which are involved in the activation of proinflammatory cytokines [149]. Furthermore, the drawbacks of viral vectors such as immunogenicity, inflammatory reactions, and low loading capacity restrict their use in miR delivery and make it challenging to establish large-scale production and quality control [150]. To get beyond the limitations of viral vectors and to inhibit microglial activation, nanometer-sized membrane vesicle-mediated miR-124 delivery has been introduced. Dendritic cells are interpreted by their ability to serve as immune cells and by their propensity to present potential antigens. EVs produced from dendritic cells (DEX-124) include a range of surface adhesion membrane proteins that facilitate efficient targeting and docking to destination cells [151]. miR-124 can be functionally delivered to microglia by DEX, which then inhibits the production of TLR4 and STAT3, two important proinflammatory mediators. DEX-124 is delivered to microglia through the nasal cavity and significantly reduced microglial activation, which in turn decreased the transcription of TLR4 and other miR-124 target genes in vivo [138]. So, preventing key PD manifestations like microglial activation and the accompanying neuroinflammation through miR-mediated mechanisms may be important therapeutic approaches in the future for PD management. Endogenous miRs are among the main problems with donor cells (DEX-124). It has been discovered that recipient cells’ biological functioning is influenced by endogenous miRs in EVs in a number of different ways [152]. Therefore, it is imperative to employ a strategy that involves the removal of endogenous miRs from the donor EVs. It has been demonstrated that dicer knockdown in donor cells diminishes the presence of miRNAs in the EVs that are recovered from these Dicer knockout cells [153]. So, this technique was employed to deplete endogenous miRs, which involved co-transfecting mouse dendritic cells with Dicer siRNA and the RVG-Lamp2b plasmid. Purified engineered EVs were then generated, loaded with either Cy5-miR-124 or Cy5-scrambled miR, and given to mouse primary microglia (mPm) and intranasally to the mouse brain [138] (indicated in Fig. 4).

Although viral vectors could deliver large levels of miR, their potential for translation is limited by safety concerns such as immunogenicity and the possibility of inducing neoplastic transformation [154]. Polymeric nanoparticle (NP)-based delivery systems, which are versatile and generally safe, successfully circumvent the challenges that viral vectors meet by being designed properly and modified appropriately and delivering miRs effectively [155–157]. The development of biocompatible and trackable polymeric nanoparticles (NPs) loaded with perfluoro-1,5-crown ether (PFCE) and shielded with protamine sulfate to complex miR-124 and its successful delivery by intracerebroventricular (i.c.v.) route to the striatum greatly increases endogenous neurogenesis in the subventricular zone (SVZ) and ameliorates PD-related motor deficits [158]. By using magnetic resonance imaging, perfluoro-1,5-crown ether aids in the non-invasive in vivo monitoring of NPs. In addition to that, poly lactic acid-co-glycolic acid (PLGA) and protamine sulfate, a cationic peptide that promotes the efficient complexation of negatively charged molecules, such as miRs. However, i.c.v.-injected polymeric NPs stay in the SVZ and line the lateral ventricles because they are unable to go to the SN. Henceforth, there are restrictions on how miRs can be delivered to DA neurons using polymeric delivery vehicles [158] (indicated in Fig. 4).

### Brain-Drug Delivery Systems to Target miR-124 in HD

The EV-focused delivery has been shown to have a substantial neuro-protective impact, which was evident from numerous articles. Exosomes from HD patient fibroblasts were injected into the ventricles of a newborn mouse brain, and this prompted HD and behavior to develop [159]. HD mice demonstrated a significant drop in the proportion of mHTT aggregates after striatal-specific infusion of astrocytic EVs [160]. A very helpful discovery was made using EVs produced from ADSC-Exos, also referred to as adipose-derived stem cells. The density of mHTT is reduced by these EVs. However, by releasing essential neurotrophic factors, it may have the ability to prevent mitochondrial dysfunction and avert cell death in an in vitro HD model, offering a promising promise for an in vivo HD therapeutic approach [161]. It is fascinating to note that the beneficial synaptic chaperone cysteine string protein (CSP) substantially eliminates the proportion of poly Q expanded (72Q) huntingtin exon 1 via EVs. The CSP may therefore aid the upkeep of protein homeostasis distinctly at synapses and the removal of mHTT from cells [162]. Even though EVs and EV-mediated therapeutic approaches have received little attention from research, miR administration utilizing EVs for HD treatment is still in its infancy. Striatum-directed Exo-124 (EVs enriched with miR-124) administration was primarily responsible for the drastically increased levels of miR-124 expression in HD mice, coupled with the concurrent suppression of REST transcription. On the other hand, the Exo-124 treatment had little effect on behavioral recovery in the HD animal model. However, it is fascinating to observe how miR-124 decreases boost REST target BDNF in HD [49] (indicated in Fig. 4 and Table 1).

**Table 1:** Modern techniques for delivering miRs to the brain to prevent AD, PD, and HD

Recent advances in miR delivery systems	Features	Mechanism	Disease	Reference
DNA nanoflowers	Extended miR-124 circulation in vivo, enhance BBB penetration and aim at neurons	MiR-124 was delivered by DFs, which decreased BACE1 and APP and stopped the production of Aβ in the hippocampi of APP/PS1 mice	AD	[76]
Upregulated miR-124-3p in microglial exosomes (Exo-124)	Intravenous injection of microglial exosomes has been taken by hippocampal neurons	Through a novel, RELA-APOE route, Exo-124 reduces the detrimental effects of Aβ and expedites the reversal of neurodegeneration	AD	[87]
Changes in the miR-132-3p level in the hippocampus of APP/PS1 mice	Overexpression of miR-132-3p via lentivirus	MiR-132-3p exhibits elevated expression in the hippocampus of APP/PS1 mice, which lowers Aβ burden and improves spatial memory impairments	AD	[143]
Small extracellular vesicles (sEVs) formed by neurons carrying miR-132	Injected via the tail vein of APP/PS1 mice and reaches brain	miR-132 expression was upregulated in GABA neurons, it was downregulated in glutamate-treated neurons The sEVs generated from neurons administered glutamate increased Aβ toxicity whereas those derived from neurons treated with GABA mitigated the harm brought on by Aβ	AD	[145]
WGA-NPs-miR-132	Administered into APP/PS1 mice via the tail vein and reaches brain	In the AD mouse model, synaptic protein levels substantially raised following treatment	AD	[147]
EVs produced from dendritic cells (DEX-124)	Through the nasal passage, miR-124 can be effectively administered to microglia	Restricts STAT3 and TLR4 production and other miR-124 target gene transcription in vivo by DEX-124, which in turn lowers microglial activation	Useful to NDDs triggered by microglial activation	[138]
Biocompatible and trackable polymeric nanoparticles (NPs) loaded with perfluoro-1,5-crown ether (PFCE) and shielded with protamine sulfate to complex miR-124	Intracerebral injection	Lessens PD-related motor impairments and positively impacts endogenous neurogenesis in the subventricular zone (SVZ)	PD	[158]
EVs enriched with miR-124 (Exo-124)	Exo-124 was injected into HD mice’s striatum on both sides	Transcription of REST is suppressed. On the other hand, the behavioral recovery in the HD animal model was not significantly impacted by the Exo-124 treatment	HD	[49]

## Conclusion and Perspectives

Early neural stem cells and neural progenitors have higher amounts of REST, which has led to its perception as a crucial transcription factor (TF) in the brain. The latest evidence, though, has astonishingly emphasized both neuron-beneficial and neurodegenerative aspects of REST in AD, PD, and HD. Despite this, certain essential miRs, such as miR-124, 132, and 9, were discovered to have a significant role in modulating REST and concurrent disease control among these diseases. However, future studies are warranted to characterize the molecular mechanisms of REST-miRs interplay in neurodegeneration. The creation of new tools, particularly those that outline how miRs should be tuned to avert NDD consequences, has emerged and will be at the heart of such future efforts. We take a glance ahead to the progression of techniques that enable the precise targeting of miRs and subsequent modification of NDD to reveal the precise mechanism of this comorbidity progression. It is challenging to send miRs to the brain because miRs must avoid immune cell phagocytosis, endonuclease degradation, the formation of serum protein aggregates, and kidney filtration in order to travel through the bloodstream and reach the target tissue. Other challenges include the instability of miRs and the reproducibility of results in miR preparations. The major drawbacks of miR delivery are those that arise from distribution across the BBB [163]. A low sensitivity range, no specificity, and dosage difficulties are the disadvantages. Because of their very short sequences and usually low copy counts, techniques used to directly identify them commonly have inadequate sensitivity. Even though there is presently no gold standard for quantifying miR expression, oligonucleotide microarray (microchip) and quantitative real-time reverse transcription PCR (qRT-PCR) are two of the most prominent methods for evaluating known miRs [164–166]. Several clinical studies are documented for miR biomarkers, particularly phase 4 trials that monitored specific miRs as biomarkers for the development of sickness in patients taking FDA-approved drugs. However, clinical research on critical NDDs is still scarce, maybe due to the constraints of miRs’ transport to the brain. BBB bridging and delivery via modified micelles, liposomes, nanoparticles, intranasal, and other delivery techniques have all been tested with varied degrees of success. In 2019, Regulus introduced RGLS5579, a novel miRNA that targets miR-10b for future clinical trials in patients with glioblastoma multiforme [167] and offers the prospect of brain targeting with miRs for NDDs.

## Data Availability

Not applicable for the article
